# Visual brain plasticity induced by central and peripheral visual field loss

**DOI:** 10.1007/s00429-018-1700-7

**Published:** 2018-06-23

**Authors:** Nicolae Sanda, Leonardo Cerliani, Colas N. Authié, Norman Sabbah, José-Alain Sahel, Christophe Habas, Avinoam B. Safran, Michel Thiebaut de Schotten

**Affiliations:** 1 Sorbonne Universités, UPMC Université Paris 06, UMR S968, Institut de la Vision, 75012 Paris, France; 2 INSERM, U968, Institut de la Vision, 75012 Paris, France; 3 CNRS, UMR 7210, Institut de la Vision, 75012 Paris, France; 4 Centre d’investigation clinique, Centre Hospitalier National d’Ophtalmologie des Quinze-Vingts, INSERM-DHOS CIC 1423, 75012 Paris, France; 5 Department of Clinical Neurosciences, Geneva University Hospital and Geneva University School of Medicine, Gabrielle-Perret-Gentil 4, 1205 Geneva, Switzerland; 6 Frontlab, UPMC Univ Paris 06, Inserm, CNRS, Institut du cerveau et la moelle (ICM), Hôpital Pitié-Salpêtrière, Boulevard de l’hôpital, 75013 Paris, France; 7 Brain Connectivity and Behaviour Group, Sorbonne University, Paris, France; 8 Department of Psychiatry, Academic Medical Centre, Amsterdam, The Netherlands; 9 Amsterdam Brain and Cognition, University of Amsterdam, Amsterdam, The Netherlands; 10 Centre de Neuroimagerie, Centre Hospitalier National d’Ophtalmologie des Quinze-Vingts, 75012 Paris, France; 11 Institute of Ophthalmology, University College of London, London, UK; 12 Fondation Ophtalmologique Adolphe de Rothschild, Paris, France; 13 Department of Ophthalmology, School of Medicine, University of Pittsburg, Pittsburg, USA; 14 Groupe d’Imagerie Neurofonctionnelle, Institut des Maladies Neurodégénératives, UMR 5293, CNRS, CEA University of Bordeaux, Bordeaux, France

**Keywords:** Visual plasticity, Cortical thickness, Resting-state cortical entropy, Central visual field loss, Peripheral visual field loss, Retinitis pigmentosa, Stargardt macular degeneration, Cytoarchitectonic areas

## Abstract

Disorders that specifically affect central and peripheral vision constitute invaluable models to study how the human brain adapts to visual deafferentation. We explored cortical changes after the loss of central or peripheral vision. Cortical thickness (CoTks) and resting-state cortical entropy (*rs-*CoEn), as a surrogate for neural and synaptic complexity, were extracted in 12 Stargardt macular dystrophy, 12 retinitis pigmentosa (tunnel vision stage), and 14 normally sighted subjects. When compared to controls, both groups with visual loss exhibited decreased CoTks in dorsal area V3d. Peripheral visual field loss also showed a specific CoTks decrease in early visual cortex and ventral area V4, while central visual field loss in dorsal area V3A. Only central visual field loss exhibited increased CoEn in LO-2 area and FG1. Current results revealed biomarkers of brain plasticity within the dorsal and the ventral visual streams following central and peripheral visual field defects.

**Electronic supplementary material:**

The online version of this article (10.1007/s00429-018-1700-7) contains supplementary material, which is available to authorized users.

## Introduction

Vision represents the most elaborated sensory input in the human brain. Central vision is captured at retinal level by the macula, which samples about 20° of the central visual field and provides a high spatial resolution. The peripheral visual field is collected by the remaining of the retina and has a low spatial resolution. The distinction between central and peripheral vision is also maintained within the brain. Particularly, dorsal visual areas receive relatively more projections from areas processing peripheral visual field representations, whereas ventral visual areas are more densely connected to those processing central representations (Ungerleider and Desimone 1986; Gattass et al. 2005). Hence, the loss of central or peripheral visual field should impair in different ways the visual brain and its neuroanatomy. Yet, little is known about the anatomical consequences and compensation mechanisms occurring after central or peripheral visual deprivation.

Central visual field loss prevents the central fixation, compelling patients to employ strategies of fixation in the peripheral retina, near the limit of the field defect (Duret et al. 1999). While the rest of visual field allows for an appropriate spatial orientation and navigation, its low spatial resolution impairs drastically object, face recognition and reading (Safran et al. 1999; Boucart et al. 2010). Reversely, peripheral visual field loss excludes the use of covert visual attention, constraining the affected individuals to increase their saccade rate to laboriously explore their environment (Authié et al. 2017). Affected individuals preserve functions related to the high spatial resolution of the residual central vision such as face and small objects recognition but exhibit impaired spatial orientation (Wittich et al. 2011) and scene perception (Fortenbaugh et al. 2007), altered postural control (Berencsi et al. 2005) and increased risk of object collision during locomotion (Turano et al. 1999, 2002) due to the limited coverage of the residual visual field. However, shreds of evidences support a brain reorganisation consequent to the adjustment of these behaviours.

Preliminary findings suggest that following a visual defect, the deafferented primary visual cortex alters its connections and the residual afferented primary visual cortex reinforces preexistent functional connections (Sabbah et al. 2017). The latter is presumably an attempt to compensate for the loss of the former to sustain higher order visual mechanisms. As central or peripheral visual loss generates sensory deprivation in a part of the visual cortex, structural alterations are also expected. Indeed, previous reports noted grey matter thinning in the posterior part of the primary visual cortex induced by central visual field loss (Boucard et al. 2009; Plank et al. 2011; Hernowo et al. 2014; Prins et al. 2016), whereas peripheral visual field loss induced thinning of the anterior part of the primary visual cortex (Boucard et al. 2009; Yu et al. 2013). However, above-mentioned studies evaluated disorders such as glaucoma and age-related macular degeneration that are not limited to the eye but imply widespread neurodegenerative cerebral alterations (McKinnon 2003; Pham et al. 2006; Woo et al. 2012; Chen et al. 2013; Cheung and Wong 2014). Such approaches may have hampered the identification of differences strictly related to early visual deafferentation and further research on disorders restricted to the retina may clarify the brain modification occurring after a pure early visual deprivation.

In the current study, we investigated long-term brain changes associated with two well-described pure and progressive retinal disorders that induce bilateral, converse visual field defects—Stargardt macular degeneration for central visual loss and non-syndromic retinitis pigmentosa for peripheral visual field loss. Stargardt macular degeneration is a hereditary cone-rod dystrophy that, in advanced stages, destroys the macular region, constraining affected individuals to rely only on the peripheral vision (Stargardt 1909). Reversely, retinitis pigmentosa—a rod-cone dystrophy—primarily affects peripheral retina and results in a progressive constriction of the visual field. It leads to a “tunnel vision” stage, with retained central vision, and later, in the most advanced stage, to blindness (Donders 1857; Sahel et al. 2015).

To evaluate the effects of the remote loss of central or peripheral vision, we estimated the cortical morphology derived from measures of cortical thickness (Das et al. 2009). However, the specific cellular mechanisms underlying the variations in cortical thickness remain obscure. They may be related to neuronal apoptosis, variations in cortical myelination, alterations of the synaptic complexity or a summation of these events (Wagstyl et al. 2015; Zilles and Amunts 2015; Burge et al. 2016). To explain the eventual differences in cortical thickness, we measured the functional MRI entropy during resting state session (*rs-*CoEn). *rs-*CoEn is a method derived from information theory and linked to neural and synaptic complexity (Tononi 1998). According to this approach, increased entropy corresponds to higher connective properties (Sokunbi et al. 2011; Yao et al. 2013; Thiebaut de Schotten et al. 2016), while the reduction in entropy may imply synaptic and dendritic degeneration (Sokunbi et al. 2013). To intimately respect its anatomy, the occipital lobe was partitioned in its corresponding cytoarchitectonic regions using cytoarchitectonic probability maps (Amunts et al. 2000; Malikovic et al. 2007; Rottschy et al. 2007; Mohlberg et al. 2012; Caspers et al. 2013; Kujovic et al. 2013; Lorenz et al. 2015; Rosenke et al. 2017). CoTks and *rs-*CoEn extracted from each of these regions were used to perform group comparisons.

## Methods and materials

### Participants

The Ethics Committee (Comité de protection des personnes, Ile de France V, and Agence Nationale de Sécurité du Médicament et des Produits de Santé) approved the study protocol (number 12,873). Thirty-eight subjects gave their written informed consent prior to inclusion. Twelve subjects suffered from Stargardt macular dystrophy (SMD) (six women, six men; all right-handed, age range from 18 to 58 year-old, mean 38.4 ± 12, median 39). This group presented a central scotoma, 10°–20° in diameter (as evaluated by Goldmann III/4 kinetic perimetry), without foveal sparing, with a best-corrected visual acuity equal or superior to 20/400 (measured by EDTRS charts). Twelve subjects suffered from retinitis pigmentosa, tunnel vision stage (RPTV) (six women, six men; nine right-handed, age range from 18 to 62 year-old, mean 41.7 ± 16.7, median 40), and presented a central residual visual field limited to a 10°–20° diameter (as evaluated by Goldmann III/4 kinetic perimetry) with a best-corrected visual acuity equal or superior to 20/40 (measured by EDTRS charts). Additionally, fourteen normally sighted controls (seven women, seven men; all right-handed, age range from 18 to 59 year-old, 41.6 ± 14.6, median 41), with normal routine ophthalmological examinations were also recruited for this study. Groups were matched for age and no significant difference between groups was observed (Kruskal–Wallis, c2(2) = 0.445, *p* = 0.801, mean rank age 19.83 in SMD group, 20.79 in RPTV group and 19.82 in normally sighted group) (see for details Supplementary Table 1).

### Neuroimaging

MRI was performed with a whole-body 3T clinical imager (Sigma Horizon) using an 8-channel head coil.

T1-weighted gradient-echo images were acquired with the following parameters TE/TR/flip angle, 3.9/9.5 ms/20°; FOV, 25.6 × 25.6 mm; matrix, 512 × 512; source voxel size, 1.2 × 0.5 × 0.5 mm; thickness, 1.2 mm, no gap.

Additionally, 32 contiguous axial T2*-weighted gradient-echo echo-planar images (TE/TR, 93/3000 ms; FOV, 240 × 240 mm; matrix, 64 × 64; voxel size, 4 × 3.75 × 3.75 mm converted to 3 × 3 × 3 mm; thickness, 4 mm; no gap; NEX, 1) were recorded to encompass the entire brain. 184 volumes were acquired including 4 “dummy” volumes obtained at the start of the session. Scan duration was 9.25 min for the whole sequence. No explicit task was required, and subjects were instructed to keep their eyes closed.

### Cortical thickness analysis (CoTks)

A registration-based method (Diffeomorphic Registration based Cortical Thickness, DiReCT) was employed to estimate the cortical thickness (Das et al. 2009) from the T1-weighted imaging dataset. The first step of this method consists in creating two-voxel thick sheets, one that lies just between the grey matter and the subcortical white matter and a second lying between the grey matter and the pia matter. Then, the former is expanded to the latter using diffeomorphic deformation estimated with ANTs (Avants et al. 2007; Klein et al. 2009; Tustison and Avants 2013). The registration produces a correspondence field that allows an estimate of the distance between the outer and inner boundaries of the grey matter ribbon, and thus cortical thickness. This approach has good scan-rescan repeatability and good neurobiological validity as it can predict, with high statistical power the age and gender of the participants (Tustison et al. 2014). All these steps were carried on automatically using BCBtoolkit (http://toolkit.bcblab.com) (Foulon et al. 2018). Average cortical thickness of the occipital lobes of each subject was also measured to account for the inter-individual variability (Ferreira et al. 2017).

### Entropy analysis (*rs-*CoEn)

First, T1-weighted gradient-echo images were skull stripped using Brain Extraction Tool (BET) as part of the FMRIB software package (FSL, http://fsl.fmrib.ox.ac.uk). T2*-weighted images were subsequently registered to the anatomical (T1-weighted) image using affine deformations. Skull-stripped T1-weighted gradient-echo images were registered to the MNI152 template (http://nist.mni.mcgill.ca/?p=904) using affine and diffeomorphic deformations (http://stnava.github.io/ANTs) (Klein et al. 2009; Avants et al. 2011). The latter deformations were applied to the T1 registered T2*-weighted images. Since the resting-state *f*MRI signal can be heavily affected by motion, even following motion correction between temporally adjacent volumes (Van Dijk et al. 2012), we estimated the signal fluctuation associated with motion and regressed it out from the *f*MRI data prior to the calculation of entropy. To this aim, we employed a recently developed and validated procedure based on data-driven Independent Component Analysis (ICA), termed ICA-Aroma (Pruim et al. 2015). This method performs an ICA decomposition of the data and estimates which components reflect motion-related noise in the *f*MRI signal on the basis of a robust set of spatial and temporal features. This is made possible due the distinctiveness of the motion-related components isolated by ICA on the *f*MRI signal (Salimi-Khorshidi et al. 2014). This approach outperforms other methods such as the regression of the motion parameter estimates, while limiting in the same time the loss in degrees of freedom (Pruim et al. 2015). Compared to spike removal methods such as scrubbing (Power et al. 2012), ICA-Aroma has the advantage of preserving the temporal structure of the *f*MRI signal. Finally, the resting-state cortical entropy (*rs-*CoEn) was estimated using FSL fslstats by first binning—using a fixed amount of 1000 bins—the pre-processed *f*MRI signal within each region of interest (ROI), and subsequently estimating the mean Shannon entropy over the entire ROI. Like in the case of CoTks, to account for inter-individual variability, we extracted the average cortical entropy for each subject. These values were estimated on a grey matter mask from the MNI single subject template, in turn obtained from FAST segmentation, and subsequent thresholding at 0.4 the partial volume estimate map of the grey matter.

### Regions of interest

To intimately respect the anatomy and cytoarchitecture of the cerebral cortex, we used probabilistic cytoarchitectonic maps of the occipital lobe (Amunts et al. 2007; Zilles and Amunts 2010; Mohlberg et al. 2012) to extract region-specific measures of cortical thickness and entropy. These regions included hOc1 (V1), hOc2 (V2), hOc3d (V3d), hOc4d (V3A), hOc3v (VP/V3v), hOc4v (V4/V4v), hOc5 (V5/ hMT+), hOc4la (LO-2), hOc4lp (LO-1), FG1, FG2 and FG4 (http://www.fz-juelich.de/inm/inm- 1/EN/Forschung/_docs/SPMAnatomyToolbox/SPMAnatomyToolbox_node.html).

### Statistical analysis

We confirmed the Gaussian distribution of the data for the three groups using the Shapiro–Wilk test (Shapiro and Wilk 1965), as well as the homogeneity of variance with the Levene test (Levene 1960).

Statistical analysis was performed with SPSS 20 (SPSS, Chicago, IL, USA). Two consecutive repeated measures ANOVAs were employed to assess differences in CoTks and *rs-*CoEn between the three groups. Cytoarchitectonic areas were considered as between subject factors and hemisphere as within subject factor. Post-hoc independent-sample t tests (Bonferroni corrected for multiple comparisons) were performed when statistically appropriate.

The relation between age, visual deficit duration and the cortical thickness and cortical entropy for the entire brain and for each cytoarchitectonic ROI was analysed through linear regression in SPSS 20 (SPSS, Chicago, IL, USA).

## Results

### Cortical thickness analysis

Whole brain average CoTks was measured for each patient and employed in group analysis, ANOVA showing no significant group effect [*F*(2, 35) = 0.557, *p* = 0.578]. Further, the measured CoTks of occipital lobe cytoarchitectonic areas were normalized with the average CoTks of the entire brain. The ratio between each cytoarchitectonic area CoTks and brain’s average CoTks was further employed in the subsequent analysis (Ferreira et al. 2017). ANOVA revealed a significant group effect for the cytoarchitectonic areas hOc1 [*F*(2, 35) = 3.882, *p* = 0.03], hOc2 [*F*(2, 35) = 4.05, *p* = 0.026], hOc3d [*F*(2, 35) = 7.09; *p* = 0.003], hOc4d [*F*(2, 35) = 3.633; *p* = 0.037], hOc4v [*F*(2, 35) = 5.013; *p* = 0.012]. Left hemispheric regions did not differ significantly from right hemisphere regions [occipital LH, *F*(2, 35) = 2.873, *p* = 0.07; occipital RH, *F*(2, 35) = 2.716; *p* = 0.08]. Post-hoc independent-sample t tests (Bonferroni corrected for multiple comparisons) are summarized in Table 1 and in the following paragraphs.

**Table 1:** Cytoarchitectonic areas showing significant differences in cortical thickness

Region	Mean CoTks ratio ± σ			ANOVA			Multiple comparisons (Bonferroni corrected)				
	Groups						NS > cVfL		NS > pVfL		cVfl > pVfL
	NS	cVfL	pVfL	*df*	*F*	*p*	*p*	Mean difference	*p*	Mean difference	*p*
Occipital RH	0.938 ± 0.054	0.899 ± 0.484	0.887 ± 0.07	2, 35	2.716	0.08	–	–	–	–	–
Occipital LH	0.916 ± 0.074	0.891 ± 0.072	0.85 ± 0.062	2, 35	2.873	0.07	–	–	–	–	–
hOc1	0.749 ± 0.113	0.652 ± 0.104	0.619 ± 0.152	2, 35	3.882	0.03	–	–	0.036	0.13 + 0.49	–
hOc2	0.847 ± 0.132	0.773 ± 0.162	0.68 ± 0.155	2, 35	4.051	0.026	–	–	0.022	0.167 ± 0.58	–
hOc3d	0.835 ± 0.093	0.707 ± 0.084	0.722 ± 0.109	2, 35	7.09	0.003	0.005	0.128 ± 0.37	0.015	0.113 ± 0.376	–
hOc3v	0.766 ± 0.118	0.704 ± 0.147	0.646 ± 0.125	2, 35	2.794	0.075	–	–	–	–	–
hOc4d	0.817 ± 0.122	0.715 ± 0.094	0.736 ± 0.083	2, 35	3.633	0.037	0.048	0.102 ± 0.04	–	–	–
hOc4v	1.072 + 0.075	0.959 ± 0.124	0.934 ± 0.153	2, 35	5.013	0.012	–	–	0.017	0.138 ± 0.047	–
hOc4la	1.155 ± 0.105	1.184 ± 0.12	1.106 ± 0.1	2, 35	1.612	0.214	–	–	–	–	–
hOc4lp	1.181 ± 0.19	1.083 ± 0.145	1.134 ± 0.146	2, 35	1.172	0.322	–	–	–	–	–
hOc5	0.976 ± 0.198	1.039 ± 0.128	0.876 ± 0.156	2, 35	2.975	0.064	–	–	–	–	–
FG1	1.253 ± 0.223	1.179 ± 0.185	1.198 ± 0.225	2, 35	0.43	0.654	–	–	–	–	–
FG2	1.278 ± 0.182	1.282 ± 0.087	1.233 ± 0.19	2, 35	0.336	0.717	–	–	–	–	–
FG3	1.362 ± 0.195	1.483 ± 0.203	1.409 + 0.266	2, 35	0.971	0.389	–	–	–	–	–
FG4	1.597 ± 0.192	1.591 ± 0.107	1.52 ± 0.17	2, 35	0.852	0.435	–	–	–	–	–

*NS* normally sighted, *cVfL* central visual field loss (SMD), *pVfL* peripheral visual field loss (RPTV)

### Visual brain CoTks in visual field loss compared to normally sighted

**Fig. 1:**
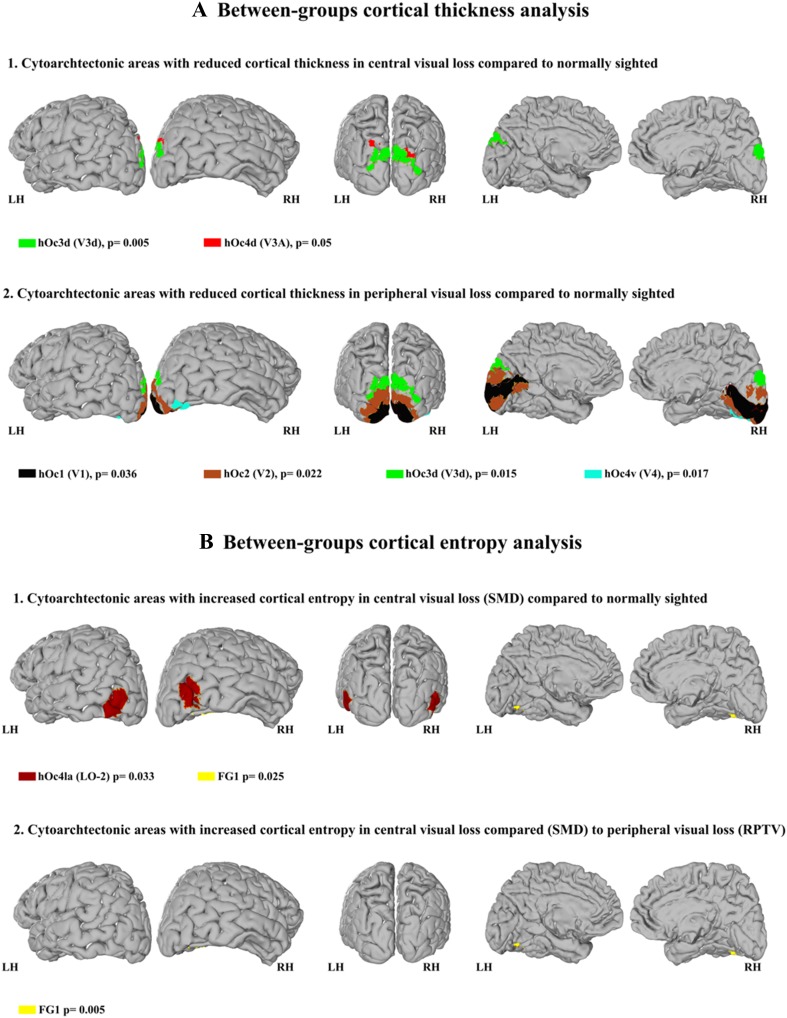
**a** Between-group analysis of cortical thickness. **b** Between-group analysis of cortical entropy. The cytoarchitectonic areas are shown on neutral brains (left hemisphere) extracted from JuBrain CytoViewer Atlas (https://www.jubrain.fz-juelich.de); each cytoarchitectonic group is depicted in a different colour

All the following post-hoc comparisons were Bonferroni corrected for multiple comparisons. Compared to normally sighted we found a significant reduction of CoTks in the dorsal region hOc3d for both central (*p* = 0.005) and peripheral (*p* = 0.015) visual field defects (see Table 1). In central visual field loss/ SMD, we also noted significant reduction in dorsal area hOc4d (*p* = 0.048) (see Fig. 1a1, and Table 1) and in peripheral visual field loss/ RPTV in early visual cortex [hOc1 (*p* = 0.036), hOc2 (*p* = 0.022)] and the ventral region hOc4v (*p* = 0.017) (see Fig. 1a2, Table 1 and also Supplementary Fig. 1).

### Visual brain CoTks differences in central and peripheral visual field loss

There was no CoTks difference between central and peripheral visual loss (see Table 1).

### Resting-state cortical entropy analysis

Whole brain average CoEn was measured for each patient and employed in group analysis, ANOVA showing no significant group effect [*F*(2, 35) = 1.378, *p* = 0.273]. Further, the measured *rs-*CoEn of occipital lobe cytoarchitectonic areas was normalized with the average *rs-*CoEn of the entire brain. The ratio between each cytoarchitectonic area *rs-*CoEn and brain’s average *rs-*CoEn was further employed in the subsequent analysis. ANOVA revealed a significant group effect for the cytoarchitectonic areas hOc4la (*F*(2, 35) = 3.856 ; *p* = 0.031) and FG1 (*F*(2, 35) = 6.566; *p* = 0.004). There was no significant effect for the factor hemisphere [occipital LH, *F*(2, 35) = 0.43, *p* = 0.654; occipital RH, *F*(2, 35) = 0.082; *p* = 0.922]. Note that none of the areas with altered CoTks compared to normally sighted exhibited significant *rs-*CoEn alterations. Post-hoc independent-sample *t* tests (Bonferroni corrected for multiple comparisons) are summarized in Table 2 and in the following paragraphs.

**Table 2:** Cytoarchitectonic areas showing significant differences in resting-state cortical entropy

Region	Mean CoEn ratio ± σ			ANOVA			Multiples comparisons (Bonferroni corrected)				
	Groups						NS < cVfL		NS / pVfL	cVfl > pVfL	
	NS	cVfL	pVfL	*df*	*F*	*p*	*p*	Mean difference	*p*	*p*	Mean difference
Occipital RH	1.126 ± 0.022	1.129 ± 0.017	1.128 ± 0.026	2, 35	0.082	0.922	–	–	–	–	–
Occipital LH	1.122 ± 0.021	1.129 ± 0.019	1.122 ± 0.024	2, 35	0.43	0.654	–	–	–	–	–
hOc1	1.071 ± 0.034	1.079 ± 0.019	1.071 ± 0.031	2, 35	0.367	0.696	–	–	–	–	–
hOc2	0.964 ± 0.045	0.913 ± 0.27	0.991 ± 0.043	2, 35	0.771	0.47	–	–	–	–	–
hOc3d	0.946 ± 0.079	0.944 ± 0.078	0.903 ± 0.073	2, 35	1.239	0.302	–	–	–	–	–
hOc3v	1.042 ± 0.028	1.052 ± 0.051	1.06 ± 0.0037	2, 35	0.693	0.507	–	–	–	–	–
hOc4d	0.911 ± 0.048	0.91 ± 0.106	0.839 ± 0.104	2, 35	2.664	0.084	–	–	–	–	–
hOc4v	1.1 ± 0.045	1.123 ± 0.046	1.093 ± 0.052	2, 35	1.356	0.271	–	–		–	–
hOc4la	1.117 ± 0.029	1.144 ± 0.02	1.136 ± 0.025	2, 35	3.856	0.031	0.033	0.027 ± 0.01	–	–	–
hOc4lp	1.08 ± 0.029	1.096 ± 0.03	1.095 ± 0.035	2, 35	1.242	0.301	–	–	–	–	–
hOc5	0.096 ± 0.066	1.016 ± 0.036	0.974 ± 0.064	2, 35	2.835	0.072	–	–	–	–	–
FG1	1.128 ± 0.025	1.158 ± 0.022	1.12 ± 0.033	2, 35	6.566	0.004	0.025	0.03 ± 0.011	–	0.005	0.038 ± 0.011
FG2	1.091 ± 0.06	1.1 ± 0.052	1.087 ± 0.062	2, 35	0.177	0.839	–	–	–	–	–
FG3	1.159 ± 0.027	1.166 ± 0.026	1.15 ± 0.032	2, 35	0.935	0.402	–	–	–	–	–
FG4	1.127 ± 0.008	1.136 ± 0.009	1.12 ± 0.009	2, 35	0.849	0.437	–	–	–	–	–

*NS* normally sighted, *cVfL* central visual field loss (SMD), *pVfL* peripheral visual field loss (RPTV)

### Visual brain rs-CoEn in visual field loss compared to normally sighted

Compared to normally sighted we found in central visual field defect/ SMD group a significant increase of *rs-*CoEn in areas hOc4la (*p* = 0.031) and FG1 (*p* = 0.025) (see Fig. 1b.1, Table 2 and also Supplementary Fig. 2). There was no *rs-*CoEn difference between peripheral visual loss/RPTV and the normally sighted.

### Visual brain rs-CoEn differences in central and peripheral visual field loss

Compared to the peripheral visual field loss/RPTV group, central visual field loss/SMD exhibited significantly higher *rs-*CoEn in the area FG1 (*p* = 0.005) (see Fig. 1b.2, Table 2 and also Supplementary Fig. 2).

### Impact of age and duration deficit on CoTks and *rs-*CoEn

There was no impact of age and deficit duration on the CoTks and *rs-*CoEn in areas exhibiting differences between groups (for regression differences in other areas see Supplementary Tables 1 and 2). Nevertheless, we found an impact of age (*p* < 0.0001) and disease duration (*p* < 0.0001) on global CoTks, but no impact on the *rs-*CoEn.

## Discussion

We assessed the impact of central and peripheral vision loss on the cortical morphology (i.e. cortical thickness, CoTks) and neural and synaptic complexity (i.e. *rs-f*MRI entropy, *rs-*CoEn). Three findings emerge from our work. First, compared to normally sighted both groups with visual field defects exhibited reduced CoTks in the dorsal region hOc3d; peripheral visual field defect group also presented reduced CoTks in early visual cortex (hOc1 and hOc2) and the ventral region hOc4V, while central visual field defect group in the dorsal region hO4d. Second, compared both to normally sighted and peripheral visual field defect groups, central visual field defect group showed increased *rs-*CoEn in FG1 area; also, compared with normally sighted, central visual field defect group exhibited increased *rs-*CoEn in hOc4la area. Finally, areas with altered CoTks had normal *rs-*CoEn and conversely.

### Differences in cortical thickness

Compared to normally sighted only the subjects with peripheral visual loss showed decreased CoTks in hOc1 and hOc2, which correspond to the functional regions V1 and V2 of the early visual cortex (Amunts et al. 2000). Previous studies reported a decreased CoTks in the early visual cortex for both central and peripheral visual loss (Boucard et al. 2009; Plank et al. 2011; Yu et al. 2013; Hernowo et al. 2014; Prins et al. 2016). In our study, only peripheral visual loss was associated with a thinning of the early visual cortex. This difference might be explained by the peculiarities of the retinal degeneration in retinitis pigmentosa. Retinitis pigmentosa is a pan-retinal, rod-cone degeneration and in the tunnel stage, patients exhibit not only the loss of all receptors in the peripheral retina, but also the loss of rods in central retina coupled to a more limited degeneration of central cones (translated by a reduced visual acuity, Sahel et al. 2015). Moreover, rod loss might directly impact certain photopic vision processes such as cone-driven, horizontal cell mediated surround inhibition (Szikra et al. 2014) or mesopic (dim-light) vision processes such as rod-cone or rod–rod gap-junction coupling presumed to help identifying dark objects moving through the visual field (Tsukamoto et al. 2001; Volgyi 2004; Ribelayga et al. 2008; Bloomfield and Völgyi 2009). SMD, on the other hand, associates a photoreceptor loss that is solely localized to central retina (Meunier and Puech 2012). Hence, the loss of cortical thickness in V1 and V2 we report herein, suggests that retinal degeneration in retinitis pigmentosa has a greater trophic impact on early visual areas. The loss of the peripheral vision represents the loss of an extensive visual field area and affects the output of numerous wide-field retinal informational channels (Ölveczky et al. 2003; Roska and Werblin 2003; Hosoya et al. 2005; Münch et al. 2009; Masland 2012). Yet poorly understood these channels might play an important role in the functioning of the early visual cortex.

The reduced hOc3d CoTks in both visual field defects when compared to normally sighted, suggest a comparable contribution of central and peripheral visual field to the dorsal portion of V3 (V3d), which is canonically included in the dorsal stream (Kujovic et al. 2013). Anatomical and functional data indicate that the primarily role of V3d area is the processing kinetic information (Felleman et al. 1997; Gegenfurtner et al. 1997; Rosa and Manger 2005), the extraction of kinetic contours (Zeki 2003), and 3D form (Vanduffel 2002). Moreover, V3d has the particularity that its retinotopical map represents only the lower quadrant of the visual field, while the upper quadrant is being represented in the ventral part of V3, area V3v (hOc3v, Rottschy et al. 2007; Kujovic et al. 2013). It is possible that the spatial nature of information processing in hOc3d/V3d is responsible for the decreased CoTks observed with both central and peripheral visual field loss. Indeed, the build-up of an accurate referential system, essential for functions such as stereopsis (i.e. 3D perception), requires both central vision, which provides high spatial resolution and fixation, and peripheral vision, which provides wide-field sampling (Goldstein and Clahane 1966; Luria 1971; Dessing et al. 2012). In central visual loss, the physiological foveal fixation lacks and compels to fixation in the vicinity of the visual field defect, in the residual functional periphery. These eccentric fixation loci (usually multiple) are used both for detection (Duret et al. 1999) and visuomotor coordination (Timberlake et al. 2012) and occur in different retinal positions for each eye. These peculiarities lead to an inadequate extraction of fixation disparities (Wheatstone 1962) impairing the very mechanism of stereopsis. In peripheral visual field loss, foveal vision, physiologic fixation and visual acuity are preserved, but stereopsis is nevertheless impaired through mechanisms such as a non-uniform drifting of the two eyes in the absence of the peripheral visual field superposition, the loss of fusion due to brief occlusions (i.e. eye-blinks, Fender and Julesz 1967) or “empty-field myopia” (i.e. accommodation impairment due to increased amplitude oscillation of accommodation in the absence of peripheral clues resulting in increased difficulty for detection, Whiteside 1957; Campbell et al. 1959). Hence, cortical thickness reduction of hOc3d CoTks in both visual field defects may account for the important contribution of central and peripheral visual fields to the functioning of this brain area.

Interestingly, compared to normally sighted, central visual field loss also exhibited decreased CoTks in the dorsal area hOc4d, corresponding to the functional region V3A. This area seems to be involved in the processing of kinetic and static 3D shapes (Georgieva et al. 2009), especially contour curvature (Caplovitz and Tse 2006), stereoscopic and chromatic motion (McKeefry et al. 2010; Anzai et al. 2011), perceptual stability during eye movements (Braddick et al. 2001; Fischer et al. 2012), the prediction of the visual motion (Maus et al. 2010), its structural damage commonly resulting in simultanagnosia, namely the inability to interpret complex visual displays despite the preserved capacity to recognize single objects (Coslett and Saffran 1991). Impaired fixation and stereoscopic vision in patients with central visual loss may account for the CoTks loss in this area.

Another intriguing result was the decreased CoTks in area hOc4v in peripheral field loss, when compared to normally sighted. Area hOc4, to the best of our knowledge, probably corresponds to human V4 (hV4) or at least to its ventral subdivision V4v (hV4v). The role of hV4 in colour perception is still debated (Bartolomeo et al. 2014), but its central participation in the figure-ground segmentation through the integration of multiple stimulus properties (i.e. contour, shape, texture, motion, colour, disparity) by bottom-up salience driven attentional mechanism or top-down proactive spatial or feature selection makes consensus (Reynolds and Desimone 2003; Qiu et al. 2007; Poort et al. 2012; Roe et al. 2012). The severely constricted visual field, resulting from the loss of the peripheral visual field, may limit covert visual attention and the sensory input in area hOc4 and consequently impact its CoTks.

### Differences in cortical entropy

Compared to normally sighted, central visual field loss group exhibited increased *rs-*CoEn in area hOc4la that likely corresponds to functionally defined LO-2 region (Larsson and Heeger 2006) involved in shape processing, object and face recognition, visual attention, action observation, visual tracking, spatial location discrimination, mental imagery and subjective emotional picture discrimination (Malikovic et al. 2016). The increased *rs-*CoEn in area hOc4la/ LO-2 suggests an adaptive increase in synaptic complexity points in this area, which is crucial for shape perception, figure-ground segregation and visuomotor coordination (Malikovic et al. 2016). Moreover, in a previous study exploring the resting state functional connectivity of central and peripheral V1 in the exact populations explored here, we found that in central visual field loss, afferented peripheral early visual cortex exhibited increased functional connectivity with LOC compared to the corresponding region in normally sighted (Sabbah et al. 2017). Therefore, in central visual loss, the increased *rs-*CoEn in LO-2 might be linked to the increased functional connectivity of this area with the residually afferented peripheral early visual cortex.

Subjects with central visual field loss presented increased *rs-*CoEn in area FG1 when compared to normally sighted and peripheral visual field loss participants. This area, located in the posterior part of the fusiform gyrus, medial to the middle fusiform sulcus (Caspers et al. 2013; Lorenz et al. 2015) exhibits a bias for the peripheral visual field representations. More precisely, FG1 and the anteriorly situated FG3 overlap with places, inanimate large objects and peripheral biased representations (Lorenz et al. 2015). This line of evidences suggests that the observed difference in *rs-*CoEn may relate to an enhanced peripheral visual field treatment in FG1 area to compensate for the central visual field loss. In accordance with this compensation hypothesis, peripheral early visual cortex in central visual field loss showed increased resting-state functional connectivity with fusiform gyrus compared to peripheral early visual cortex in normally sighted.

### Combined cortical thickness and cortical entropy data

Interestingly, the regions with altered CoTks had normal *rs-*CoEn and conversely. Limitations aside, reduced CoTks with normal *rs-*CoEn may indicate a possible mixture of cell shrinkage with preserved synaptic complexity of the remaining networks. On the other hand, increased *rs-*CoEn might correspond to a brain plasticity effect induced by an increased visual afference of the spared visual field.

## Limitations

Cytoarchitectonic areas are highly variable across subjects (Amunts et al. 1999). Herein, we extracted occipital cytoarchitectonic areas from an atlas based on observer-independent probabilistic mapping of ten post-mortem brains (Mohlberg et al. 2012; Zilles and Amunts 2015). To reduce the effect of inter-individual variability, CoTks and *rs-*CoEn were sampled only in voxels where the same cytoarchitectonic area overlaps in more than five out of ten of the post-mortem brains investigated.

Myelin density in the cortex and various technical parameters (field strength, tissue segmentation methods, smoothing, etc.) may influence the MRI measure of cortical thickness and lead to incorrect estimations (Glasser et al. 2014). These effects can be particularly deceptive in disorders that impact myelination (i.e. multiple sclerosis) or in physiological states exhibiting different degrees of cortical myelination (i.e. development or ageing, Westlye et al. 2010; Zilles and Amunts 2015). Unfortunately, we could not assess cortical myelination with T1-weighted images. Future research investigating T1 intensity may shed light on that matter (Turner et al. 2008; Stüber et al. 2014).

Resting-state *f*MRI signal is notoriously affected by motion (Van Dijk et al. 2012). Increased movements would virtually increase measures of entropy. To reduce this effect, we regressed out the motion-related signal from the *f*MRI data before the calculation of entropy. In this way, we maximised the likelihood that the entropy measures reflect the spontaneous hemodynamic fluctuations related to brain activity.

We noted an overall effect of age and in patient groups also of disease duration on brain CoTks, but not on the CoEn. However, we found no effect of age and disease duration on CoTks in areas exhibiting differences between groups. The lack of effect of age and disease duration on the CoTks of the studied cytoarcitectonic areas might be due to limited size of our studied groups. In addition, the absent impact of age and disease duration on CoEn might reflect the plastic changes resulting in a preserved synaptic complexity.

## Conclusion

Overall, central and peripheral visual loss induced complex structural changes unpredicted by the canonical segregation central vision—ventral visual field, peripheral vision—dorsal visual field. We found that central visual field loss induces a thinning in dorsal stream areas hOc3d (V3d) and hOc4d (V3A) and peripheral visual field loss in early visual cortex (hOc1/V1 and hOc2/V2), dorsal stream area hOc3d (V3d) and ventral stream area hOc4v (V4). Central visual field loss also induces an increase in entropy in areas hOc4la (LO-2) and FG-1 reflecting possible alternative, compensatory processing. These results offer a new and interesting insight on the effect of central and peripheral visual field deafferentation and also invite to revisit the canonical concepts of “ventral” and “dorsal” stream. Moreover, these data suggest complex adaptive changes that should be considered in the development of new visually rehabilitation strategies, sensory substitution devices or visual restitution attempts.

## Electronic supplementary material

Below is the link to the electronic supplementary material.

Between-group analysis of cortical thickness (PNG 46 KB)

Between-group analysis of cortical entropy (PNG 38 KB)

Clinical data about vision loss onset and evolution. Note that determining the real onset of retinitis pigmentosa is very challenging. Individuals become aware about the visual field defect relatively late in the disease due to fading and filling-in processes. Therefore, the recorded onset age should be regarded with caution for certain subjects. (PNG 143 KB)

Regression analysis showing the significant effects of age and visual defect duration on normalized CoTks (PNG 62 KB)

Regression analysis showing the significant effects of age and visual defect duration on normalized CoEn (PNG 59 KB)

Regression analysis showing the significant effects of age and visual defect duration on whole brain CoTks and CoEn (PNG 40 KB)
