# Supplementary figures and images for: Visual brain plasticity induced by central and peripheral visual field loss
Source: Brain Struct Funct. 2018 Jun 23;223(7):3473–85. doi: 10.1007/s00429-018-1700-7 (PMC6132657; doi:10.1007/s00429-018-1700-7)

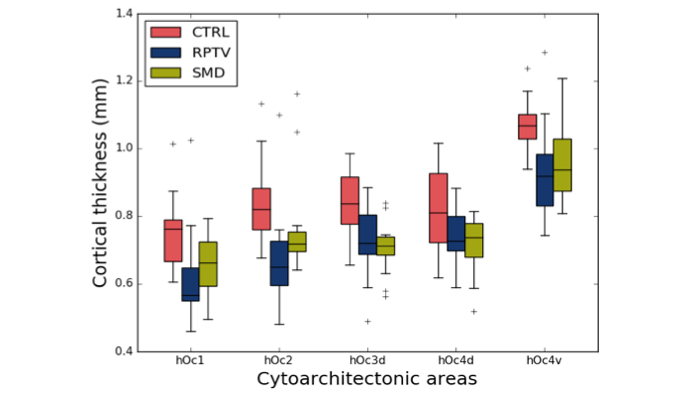

Supplement: Supplementary file 1 — Between-group analysis of cortical thickness (PNG 46 KB) [file 429_2018_1700_MOESM1_ESM.png]

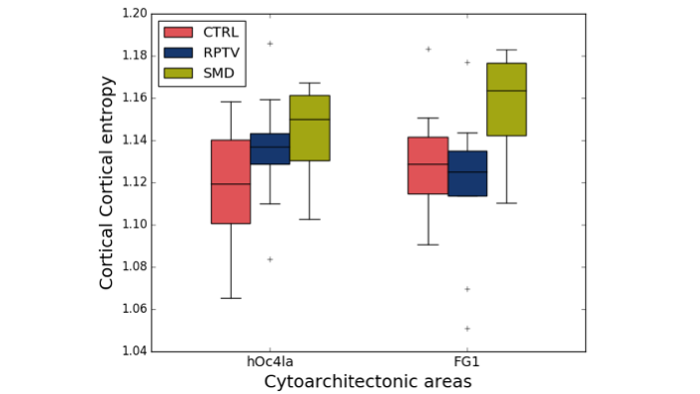

Supplement: Supplementary file 2 — Between-group analysis of cortical entropy (PNG 38 KB) [file 429_2018_1700_MOESM2_ESM.png]

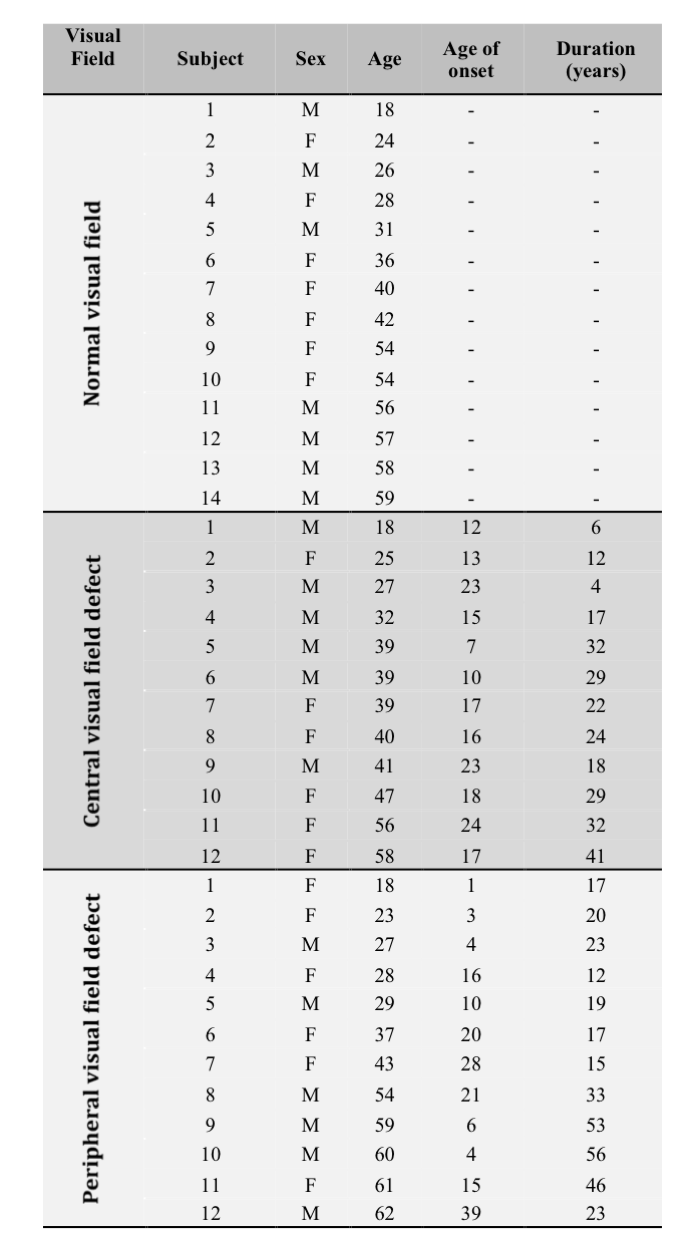

Supplement: Supplementary file 3 — Clinical data about vision loss onset and evolution. Note that determining the real onset of retinitis pigmentosa is very challenging. Individuals become aware about the visual field defect relatively late in the disease due to fading and filling-in processes. Therefore, the recorded onset age should be regarded with caution for certain subjects. (PNG 143 KB) [file 429_2018_1700_MOESM3_ESM.png]

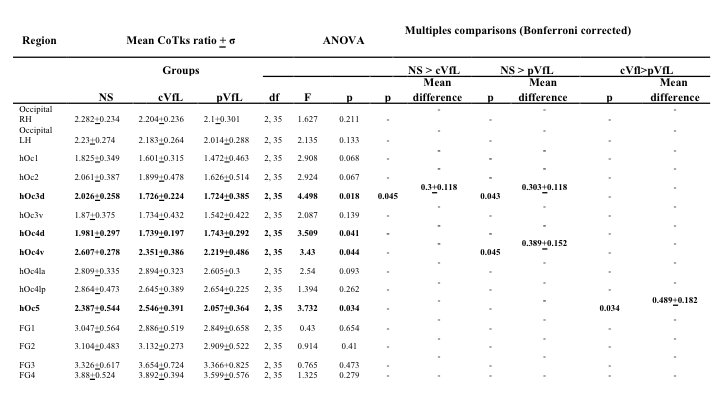

Supplement: Supplementary file 4 — Regression analysis showing the significant effects of age and visual defect duration on normalized CoTks (PNG 62 KB) [file 429_2018_1700_MOESM4_ESM.png]

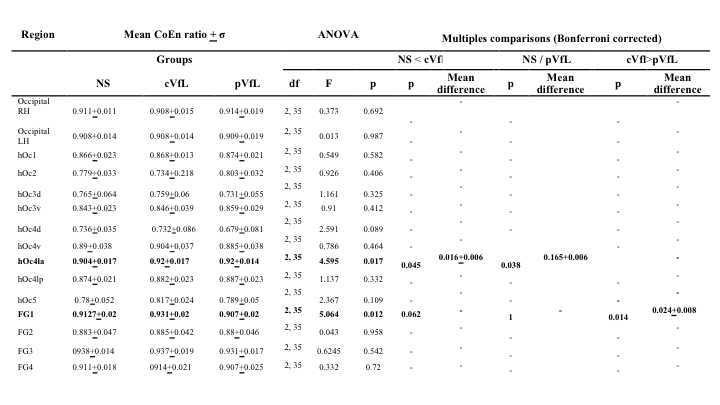

Supplement: Supplementary file 5 — Regression analysis showing the significant effects of age and visual defect duration on normalized CoEn (PNG 59 KB) [file 429_2018_1700_MOESM5_ESM.png]

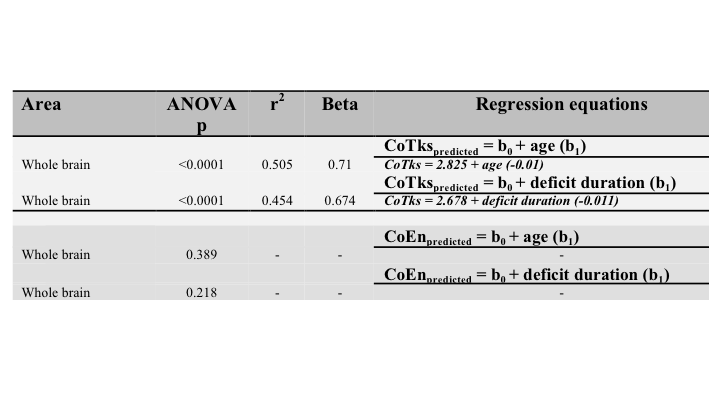

Supplement: Supplementary file 6 — Regression analysis showing the significant effects of age and visual defect duration on whole brain CoTks and CoEn (PNG 40 KB) [file 429_2018_1700_MOESM6_ESM.png]
